# Diaphragm injuries in a mature trauma system: still a diagnostic challenge

**DOI:** 10.3389/fsurg.2024.1489260

**Published:** 2024-12-09

**Authors:** S. Karhof, R. K. J. Simmermacher, P. Gerbranda, K. J. P. van Wessem, L. P. H. Leenen, F. Hietbrink

**Affiliations:** Department of Surgery, University Medical Centre Utrecht, Utrecht, Netherlands

**Keywords:** diaphragm, diaphragm injury, traumatic diaphragmatic injury, diaphragmatic hernia, abdominal trauma

## Abstract

**Background:**

A traumatic diaphragm defect is a rare injury. A missed diaphragm injury may cause serious morbidity and mortality. Detection rate during the first assessment of trauma patients is notoriously low. However, important improvements in imaging modalities were developed. The aim of this study was to analyze traumatic diaphragm injuries in relation to diagnostic tools, therapeutic interventions and outcome over the past two decades.

**Methods:**

A retrospective analysis was performed of all trauma patients with traumatic diaphragm injuries between 2000 and 2018 at a level I trauma center. Data collected were baseline characteristics, diagnostics that were performed, treatment given and follow-up.

**Results:**

A total of 47 patients with traumatic diaphragm injuries were evaluated. The majority of injuries was seen following blunt trauma (72%). Mortality was 21%, mainly due to concomitant injuries. One patient died due to the consequences of an unrecognized diaphragm injury. In 29 cases (62%) the injury was diagnosed pre-operatively through imaging, with the remaining being diagnosed during laparotomy. In 11 patients (35%) the diaphragmatic injury was not seen on a pre-operative CT-scan. Postoperative complications occurred in 19 patients, mostly of pulmonary origin (i.e., pneumonia). No recurrences were reported.

**Conclusion:**

This study confirms diaphragm injuries are infrequent injuries, with high mortality. Even more, despite major improvement in diagnostic modalities over the past 2 decades, the algorithm for detection of diaphragmatic injuries has not changed nor has its outcome. Although the incidence is low, since consequences are severe, it is important to have a high index of suspicion in abdominal trauma, even in a non-conclusive CT-scan.

## Background

Diaphragm laceration, following blunt or penetrating trauma are insidious injuries (1–6). The incidence, in literature, varies widely and depends on different factors (1–6). In the first place the trauma mechanism matters with an 2:1 ratio when comparing penetrating to blunt trauma (1). Secondly, the diagnostic pathway followed after trauma differs, leading to different incidences concerning a possible diaphragm lesion.

Currently, reported mortality in patients with traumatic diaphragmatic injury (TDI) is still up to 40% (1–6). This rather high rate is mainly due to serious concomitant injuries and not so much the diaphragm-injury itself. Nevertheless, an initially unrecognized TDI itself may come with serious complications, as a longstanding diaphragm defect might result in herniation or strangulation of displaced intra-abdominal content. This herniation may, irrespective the cause, lead to respiratory insufficiency by oppression of the thoracic content (1, 2), or digestive difficulties or even abdominal ischemia due to strangulation of the hernia content.

Injury as result of penetrating trauma does not need further explanation, as it is a direct laceration of the diaphragm muscle. Blunt trauma results in an increased intra-abdominal pressure possibly leading to a burst defect in the diaphragm. Anatomy-physiologic studies suggest a higher incidence of left-sided injuries due to congenital weakness along embryonic fusion of costal and lumbar portions of the diaphragm, mainly in blunt trauma (1). Additionally, the liver is seen as a protecting factor of the right side of the diaphragm, making clinically relevant right sided diaphragm injuries less frequent (1, 3–5).

Clinical diagnosis can be difficult since no single non-invasive diagnostic tool is sensitive or specific enough to accurately confirm this injury, with injuries often being discovered “by accident” during laparotomy for another reason (1–3, 6). This diagnostic dilemma is not new, raising the question whether anything has changed in this century concerning the diagnostic possibilities to discover a diaphragm injury before operation.

Given the nature of these injuries, literature consists mainly of case reports or small retrospective studies (case series). The purpose of this study was to add to the current available information by evaluation of our traumatic diaphragmatic injuries over the past decades and to explore whether in this era of advanced diagnostics, changes could be achieved in diagnostic tools, therapeutic interventions and (postoperative) complications.

## Material and methods

A retrospective review of a prospectively collected trauma database was performed, examining all trauma patients presenting with a possible traumatic diaphragmatic injury at our level I trauma center located in a European country with limited violence between 1st of January 2000 to the 31st of December 2018. All data concerning presentation at our trauma department were collected, including patient characteristics (age, sex and medical history), trauma mechanism, injury type, performed diagnostics, as well as treatment (timing and type of repair) and follow-up. Trauma mechanism was divided in motor vehicle accident (MVA), fall, stab wound, gunshot wound and others. Presentation was defined either as acute or delayed; all cases in which the diaphragm injury was found within the same admission following trauma were named acute, all others were called delayed. These injuries can therefore also be considered as missed injuries. In the acute setting, assessment as well as the diagnostic pathway were along ATLS® principles (7). Delayed presentation mainly consisted of referred patients in whom abdominal content already had herniated, being obvious on plain radiographs. In these cases the diagnostic algorithm used during the primary assessment was noticed.

In treatment a distinction between immediate and postponed repair was made. Immediate repair refers to surgery within 48 h following trauma, everything thereafter was defined as postponed repair. Morbidity and mortality were evaluated for all patients during the follow-up at our hospital. Complications like pneumonia or empyema, bowel obstruction, incisional hernia, urinary tract infection, delirium, re-admission and recurrence were all taken into account.

### Statistical analysis

All statistical analyses were conducted using IBM SPSS Statistics 22.0 (IBM Corporation, Armonk, NY, USA). Continuous variables were reported in medians, including ranges. Discrete variables were displayed as proportions. Univariate analysis was performed using the Chi square and Fisher's exact test. For comparisons between more than two independent groups, the Kruskal-Wallis one-way analysis of variance test was applied. *P*-values below 0.05 were considered statistically significant.

## Results

**Figure 1:**
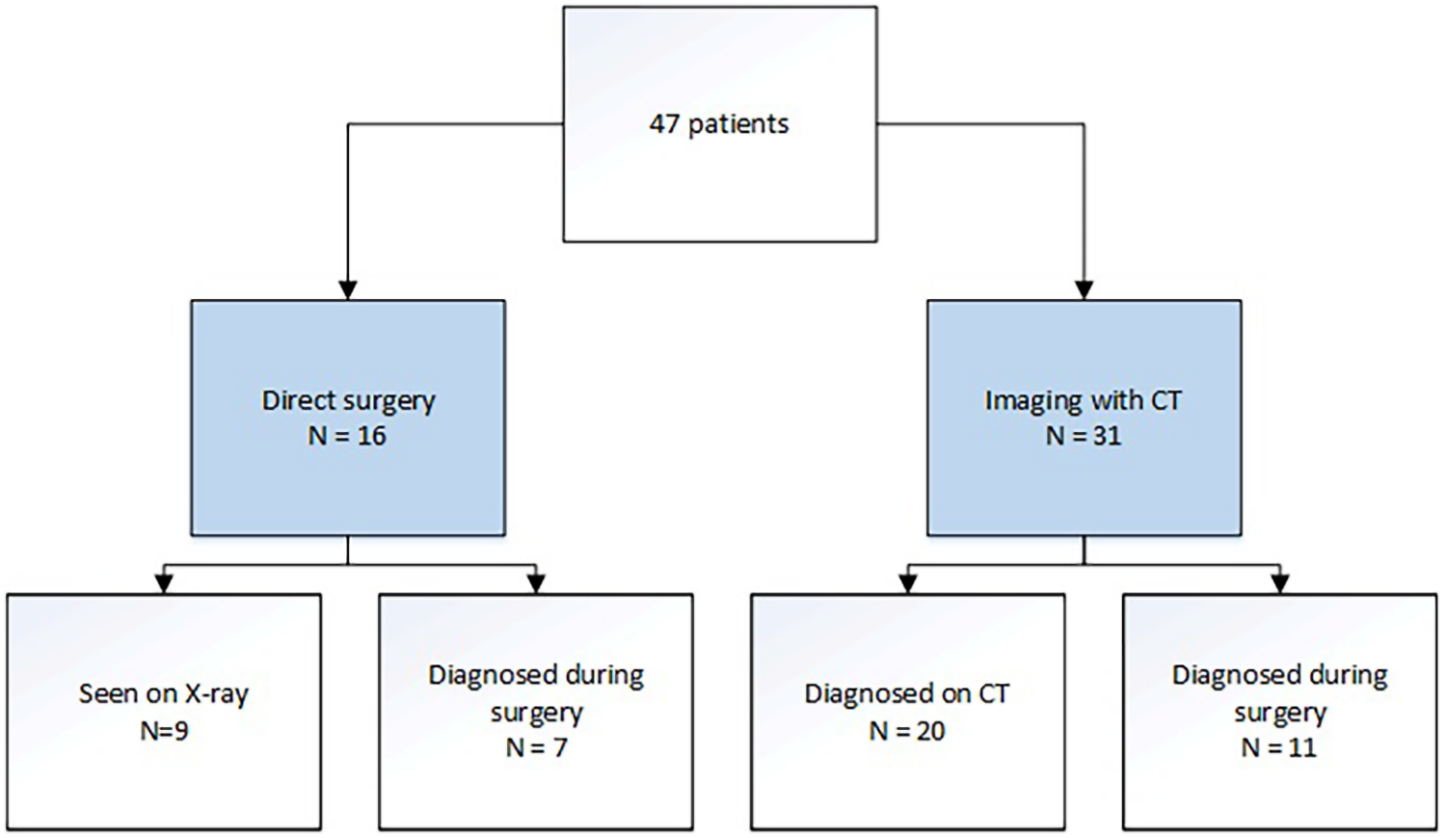
Flowchart work-up imaging.

In the nineteen years reviewed, a total of 47 patients with a traumatic diaphragmatic injury were directly presented at our level I trauma center. With a yearly average of about 1,500 trauma patients in our resuscitation bay in the emergency department, this would lead to an incidence of approximately 0.15%. Thirty-four of these injuries were caused by blunt trauma mechanisms, mainly MVA (Table 1). In one patient the mechanism of injury was rather unclear since the diaphragm rupture appeared to be relatively old during surgery: as this patient was involved in a stabbing incident several years earlier, this was probably the cause, rather than the blunt injury he presented with now. The majority of patients had left-sided TDI (31/47). Most of the diaphragmatic injuries were diagnosed non-invasively during the primary assessment (29/47) with 9 patients being diagnosed on x-ray and the remaining 20 diagnosed on CT-scan (Figure 1). Within the 18 injuries first found during laparotomy, half were due to blunt trauma mechanism (Table 1). Of all patients, a total of 31 had pre-operative chest x-ray and/or high-resolution CT-scan, in 11 of them, the diaphragm injury was not recognized before surgery (Figure 1). In 27 patients (27/48) herniation of abdominal organs had occurred, with the majority following blunt injury (25/27), most often (13/27) the stomach herniated into the thoracic cavity (Table 1). Most patients presented within the acute phase after injury, only 4 patients had a delayed presentation, varying from 4 weeks to one extremely late presentation after 17 years. Thirty-six patients received immediate diaphragmatic repair (Table 2). In 5 patients a postponed repair was performed. In two patients the diaphragm injury was not identified immediately and were repaired after 6 and 15 days respectively. Another three of them had a delayed presentation without severe complaints and were scheduled for surgery within a few days following presentation. Of all surgeries performed, 41 of the defects underwent primary repair with sutures, in 1 patient a mesh was used to close the defect (the patient who presented 17 years following trauma) and in the remaining 5 patients there was limited or no closure performed as described next. In 2 of them there was no closure due to uncontrollable hemorrhage and only packing had been performed after which both patients died before a subsequent intervention could be performed, in 1 patient the defect was small and closed with sealant, and in the other 2 it was decided not to be needed due to the small size (<1 cm) with pericardium covering the defect in one of the injuries and only a partial (thoracic) defect in the other with peritoneum covering the other side. These injuries were all caused by penetrating trauma mechanism, except for the ones with uncontrollable hemorrhage, and most of them (4/5) were on the left side of the diaphragm.

**Table 1:** Baseline characteristics.

	Total	Blunt	Penetrating	*P*-value
	*N* = 47	*N* = 34 (72)	*N* = 13 (28)	
Age in years, median (IQR)	32 (31)	29 (36)	33 (24)	0.934
Male (%)	35 (75)	23 (68)	12 (92)	0.082
Medical history				
None	32	25	7	
Psychiatric	3	2	1	
Previous trauma	3	1	2	
Cardiac	3	3	0	
Diabetes	3	1	2	
Other	2	1	1	
Trauma mechanism				
MVA	30	30	0	
Fall	3	3	0	
Stabbing	9	0	9	
Shooting	4	0	4	
Other	1	1	0	
Side of injury				
Right	14 (30)	11 (32)	3 (23)	0.404
Left	31 (66)	21 (62)	10 (77)	0.266
Bilateral	2 (4)	2 (6)	0	0.519
Presentation				
Acute	43	32	11	0.304
Delayed	4	2	2	
Size of the defect^a^, cm Median (IQR)	4 (7.5)	9 (4)	2 (2)	**0** **.** **005**
Herniation	27	25	2	**0** **.** **001**
Liver	9	9	0	**0** **.** **038**
Stomach	13	13	0	**0** **.** **007**
Small intestine	4	4	0	0.260
Large intestine	3	2	1	0.631
Omentum	6	4	2	0.538
Spleen	7	7	0	0.086
ISS median (IQR)	33 (53)	34 (19)	19 (26)	**0** **.** **004**
Diagnosis				
Imaging	29	24	4	**0** **.** **011**
During surgery	18	9	9	
Pre-operative CT^b^	11	6	5	0.500

^a^ For blunt *n* = 9, penetrating *n* = 8 (in others the size of the injury was not documented).

^b^ For the patients in which diagnosis was made during laparotomy (*n* = 18).

Bold values are significant.

**Table 2:** Treatment.

	Total	Blunt	Penetrating	Sign
	*N* = 47	*N* = 34	*N* = 13	
Timing^a^				
Immediate	36	28	8	0.073
Postponed repair	5	4	1	
Approach^a^				
Thoracotomy	7	6	1	0.391
Thoracoscopy^b^	1	0	1	
Thoraco-phrenico laparotomy	4	3	1	
Laparotomy	35	25	10	
Type of repair				
Primary	41	31	10	**0** **.** **002**
Mesh	1	1	0	
No (or limited) repair	5	2	3	
Length of stay total in days	13 (25)	15.5 (29)	9 (10)	0.150
Length of stay ICU in days	5 (11)	7 (11)	1.5 (3)	0.070
Admission ICU (%)	36 (77)	31 (91)	5 (39)	**<0** **.** **001**
Complications (%)	19 (41)	16	3	0.106
Pneumonia	11	8	3	
Empyema	2	2	0	
ARDS	1	1	0	
Bowel obstruction	2	2	0	
Incisional hernia	2	2	0	
Cardiac arrest^c^	1	1	0	
Other^d^	5	5	0	
Recurrence	0	-	-	NA
Follow-up in months	6 (17.5)	10 (27)	5 (7)	0.100
Mortality	10	10	0	**0** **.** **025**
Exsanguination	5	5	-	
Neurologic	4	4	-	
Asphyxia	1	1	-	

NA, not applicable.

^a^ Total *N* = 41, in 6 cases no (or limited) repair was performed.

^b^ In total 3 patients were started with thoracoscopic approach, however 2 of them were converted to thoracotomy or thoraco-phrenico laparotomy and therefore counted into these groups.

^c^ Following gastric and omental ischemia due to late recognized defect.

^d^ 3x Urinary tract infection, 2x delirium.

Bold values are significant.

In most cases that underwent repair, a laparotomy was performed to approach the injury (35/47). The remaining twelve were approached with a thoracic or combined approach. In 3 of these cases a thoracoscopic procedure was performed, however conversion was needed in 2/3 due to herniation. In most of the delayed repairs (4/5) a thoracic approach was preferred, although followed by a laparotomy in 2 cases.

Thirty-six of the patients, were subsequently admitted to the Intensive Care Unit (ICU) due to their concomitant injuries. Complications were reported in 19 patients (40%). Most patients suffered from pulmonary complications (Table 2). Two patients had a bowel obstruction requiring relaparotomy, 1 and 3 months following trauma, respectively. Two other patients developed an incisional hernia, both approximately 1 year after trauma. One was from a subcostal incision, which was repaired with a mesh, the other one was a midline hernia and was reconstructed by Ramirez technique (8). The most serious complication was cardiac arrest in a primarily unrecognized diaphragm defect (not detected on CT-scan). In this patient the diaphragm defect had caused a herniation of the stomach to the thoracic cavity which resulted in partial gastric necrosis and subsequent pericarditis and cardiac arrythmias followed by cardiac arrest. Recurrences of the diaphragm injury were not seen during follow-up, which was a median of 6 months, with a median time of 10 months for patients following blunt trauma and 5 months after penetrating trauma. Ten patients died during follow-up, most of them within the first 24 h following trauma (*n* = 7), due to causes unrelated to the diaphragm injury.

## Discussion

This study demonstrates that diaphragm injuries are still rather rare injuries in this European setting with limited penetrating injuries, with an incidence of 0.15% seen in a mature level one trauma center with a mortality of 21% mainly due to concomitant injuries (i.e., neurotrauma). This incidence is nearly the same as in the late decennia of the former century (9, 10). In line with the incidence of penetrating trauma in our country, most TDI were the result of blunt trauma mechanism (34/47) and more often on the left side of the diaphragm (31/47). Although the majority was diagnosed by imaging, still 11/31 (30%) of the injuries were not identified on the pre-operative CT-scan, but discovered during surgery. The accuracy in detecting diaphragm injuries continues to appear limited despite the development of more sophisticated non-invasive diagnostic tools in the past decades.

Traumatic diaphragm injuries in the Netherlands have been previously described by van Vugt et al. in 1989 (10). They reported a case series of 32 patients within 10 years, mainly (28/32) due to blunt trauma mechanism. Like our study population most of their patients were young males, with high Injury Severity Score (ISS). Although their study dates almost 30 years before our current study, the results appear to be similar. In only half of the patients the diagnosis was made before surgery, mostly with chest x-ray, CT-scan was not widely available in that period. They describe 5 patients with a delayed diagnosis of diaphragm injury (ranging from 24 h to 8 years) and most of the patients were treated by primary repair through laparotomy. Mortality was 28.6% in their study population. This incidence and outcome are similar to our more recent data.

Where most other studies have a blunt vs. penetrating ratio of at least 1:2, ours is the opposite. This is probably due to the low incidence of stab- and gunshot wounds in our country, compared to other parts of the world. This is also described in a large, recent study based on the traumaregister DGU (11). They found 687 patients with a diaphragmatic rupture in their study population of 199.933 patients from different European (mainly German) hospitals, leading to a prevalence of 0.3%. Our results are pretty much comparable to theirs, with most injuries found in young males with high ISS scores and relative high mortality, mainly due to other injuries. However, with an important difference in detection rate, where in this large database study 93% of the injuries were discovered in the resuscitation room. This is noticeably higher than in our study population, where a total of 11 diaphragm injuries were not recognized on pre-operative CT-scan (23%). Even more, 4 patients in our study presented with a diaphragm injury a long time after trauma, varying from 4 weeks to several months (with one outlier of 17 years). It might even be possible that we have missed more injuries which have not yet become symptomatic. This highlights how difficult the injury is to diagnose on imaging alone, even when compared to earlier studies within the Netherlands, where most of the injuries were diagnosed on x-ray while computed tomography was not yet widely available (10). The difference in detection rate is not completely understood, probably also since both studies have a retrospective design where a trauma database was used to collect data and missing parameters might have a huge impact. One possible explanation is that the study of Weber et al. (11) had their focus on relevant diaphragmatic ruptures (AIS ≥ 3) and smaller (irrelevant) defects might have been missed. This could also explain why all injuries were taken to the operating room.

Ties et al. compared traumatic diaphragm injuries between two time periods (1996–2003 vs. 2004–2011) (12). They found a total of 146 vs. 308 patients, most following penetrating trauma (79% vs. 73% respectively). Within both groups mortality was higher in patients following blunt trauma (15% vs. 4% in penetrating injury). Although CT-scanning was widely available in the second time period, there were no significant differences in diagnosis rates. This finding underlines the persisting diagnostic difficulty of this injury, which is easily missed even on multislice CT-scan. This is also shown in our results when compared to the results of van Vugt et al. (10). Although in our study group performance of a CT-scan was widely available diagnostic accuracy still only improved from 50% to 65% (with still 11/31 injuries missed on CT-scan). In the end, surgery clearly remains the gold standard for diagnosing a diaphragm injury.

Other recent studies also described low incidences of 0.45%–1.6% and high mortality rates 7%–27% (3, 4, 13–16) (Table 3). In most previously published studies, diaphragm injuries were the result of penetrating trauma 73%–94%. Blunt trauma leading to diaphragm injuries was found to have a significant higher ISS, length of ICU and hospital stay and a higher mortality. One study compared acute with chronic diaphragm injuries in 50 patients. Most of the injuries were due to blunt trauma (90%) and in the left diaphragm (72%). Within these patients, 19 (38%) presented with a chronic diaphragm injury with a range of 1–30 years between trauma and diagnosis (17). They found a significant difference in size of the injury with a mean size of 6 cm in the chronic presentation (compared to 11 cm in the acute presentations). Within follow-up they found one recurrence which was repaired with a mesh the second time. As far as we know, this is the only study describing a recurrence after surgical repair for a diaphragm injury.

**Table 3:** Numbers in previous studies.

	Van Vugt et al. (1989) (10)	Lewis et al. (2009) (2)	Zarour et al. (2013) (3)	Fair et al. (2015) (4)	Mahamid et al. (2017) (13)	D'Souza et al. (2017) (14)	Cardoso et al. (2017) (15)	Kaya et al. (2020) (16)
Data origin	Hospital medical records	Hospital medical records	Trauma registry	National Trauma Databank	National Trauma Registry	Hospital medical records	Hospital medical records	Hospital medical records
Total *N*	-	-	87,294	8,33,309	3,54,307	6,604	-	-
Diaphragm injuries	32	254	773	3,783	231	105	103	92
Incidence	-	-	0.8%	0.45%	0.07%	1.6%	-	-
Country	NL	USA	USA	USA	Israel	South Africa	USA	Turkey
Side of injury								
Right	-	36%	40%	-	-	21%	46%	28%
Left		60%^a^	57%^a^			79%	54%	59%^a^
Mechanism								
Blunt	91%	49%	27%	33%	100%^b^	6%	2%	23%
Penetrating	9%	61%	73%	67%		94%	98%	77%
Surgery	87.5%	-	81%	-	62%	-	87%	92%
Mortality	28.6%	22%	21%	12.4%	27%	7%	16.5%	15.2%

-, not described.

^a^ For Lewis et al. 4% and for Zarour et al. 3% of the diaphragm injuries was bilateral; for Kaya et al. 9% of the TDI were bilateral and 4% unknown.

^b^ Mahamid et al. have investigated the incidence of traumatic diaphragm injury only in blunt trauma patients.

The most important limitation in our study is the retrospective design with limited patient population and relative short median follow-up. However, it can be assumed that given the medical system in our country, patients with clinically relevant problems due to their injury would report back to our hospital. The true incidence of diaphragm injury most probably never will be known as the golden standard would be surgery in all trauma cases, especially the blunt ones (laparotomy for hemodynamically unstable patients, in other cases a diagnostic laparoscopy or thoracoscopy might be considered as well, depending on operators preference). Furthermore, we might miss out on some additional information due to its retrospective nature of the study.

## Conclusion

In conclusion, it should be realized that although the relative small numbers in this study, our results highlight what previous studies have shown before despite tremendous changes in diagnostic modalities: diaphragm injuries have a low incidence, they are difficult to diagnose and are associated with high morbidity and mortality rates. Even though diagnostic modalities continue to improve and become more readily available over the past decades, this injury remains easily missed pre-operative in a large part of the patient population. Therefore, awareness should be created in suspicious cases with a high probability as it might point to more serious, insidious, concomitant injuries with potential immediate or delayed life-threatening complications, while the injury itself demands urgent reconstruction.

## Data Availability

The data analyzed in this study is subject to the following licenses/restrictions: The datasets used and/or analysed during the current study are available from the corresponding author on reasonable request. Requests to access these datasets should be directed to steffikarhof@gmail.com.
